# Psychopathological features of hysterical disorders arising as part of affective disorders and schizophrenia

**DOI:** 10.1192/j.eurpsy.2022.1443

**Published:** 2022-09-01

**Authors:** A. Barkhatova, S. Popov, S. Sorokin

**Affiliations:** Mental Health Research Center, Department Of Endogenous Mental Disorders And Affective Conditions, Moscow, Russian Federation

**Keywords:** Hysterical disorders, conversions, depression, schizophrenia

## Abstract

**Introduction:**

Hysterical disorders were considered separately in the context of the dynamics of the course of either endogenous affective diseases or schizophrenia, without attention to the conjugation and interaction of issues of hysterical symptoms and affective or psychotic syndromes.

**Objectives:**

To test the psychopathological structure and provide a typology of the conjugation of hysterical symptoms with other psychopathological syndromes.

**Methods:**

120 patients (82 women and 38 men) with schizophrenic and affective disorders with associated hysterical symptoms were examined by a clinical psychopathological method.

**Results:**

Three variants of conjugation were identified. In the group of hysterical disorders associated with affective diseases (37,1%) the structure and dynamics of hysterical symptoms directly influenced the developing affective phase: the low intensity of hysterical symptoms contributed to the development of an apatho-adynamic type of depression, and bright and spontaneous hysterical manifestations formed an anxious-hypochondriac type of depression. Hysterical disorders formed in the structure of the psychotic state (41,4%) influenced the nature, structure, dynamics and content of delusional, hallucinatory and paranoid disorders. “Caste” hysterical symptoms (21,4%) revealed a lack of connection with affective and psychotic states. Hysterical symptoms were characterized by persistence, stability, invariability of manifestations, long-term psychotherapeutic and psychopharmacological resistance.

**Conclusions:**

Clinical and psychopathological analysis of endogenous mental diseases of the affective and schizophrenic spectrum, occurring with hysterical symptoms, showed that the parameter of the conjugation of hysterical symptoms with other psychopathological syndromes is prognostically significant.

**Disclosure:**

No significant relationships.

